# Molecular Detection of Tick-Borne Pathogen Diversities in Ticks from Livestock and Reptiles along the Shores and Adjacent Islands of Lake Victoria and Lake Baringo, Kenya

**DOI:** 10.3389/fvets.2017.00073

**Published:** 2017-06-01

**Authors:** David Omondi, Daniel K. Masiga, Burtram C. Fielding, Edward Kariuki, Yvonne Ukamaka Ajamma, Micky M. Mwamuye, Daniel O. Ouso, Jandouwe Villinger

**Affiliations:** ^1^International Centre of Insect Physiology and Ecology (icipe), Nairobi, Kenya; ^2^University of Western Cape, Bellville, South Africa; ^3^Egerton University, Egerton, Kenya; ^4^Kenya Wildlife Service, Nairobi, Kenya; ^5^Jomo Kenyatta University of Agriculture and Technology, Nairobi, Kenya

**Keywords:** tick-borne diseases, *Ehrlichia*, *Anaplasma*, *Rickettsia*, *Babesia*, *Hepatozoon*, *Theileria*, Kenya

## Abstract

Although diverse tick-borne pathogens (TBPs) are endemic to East Africa, with recognized impact on human and livestock health, their diversity and specific interactions with tick and vertebrate host species remain poorly understood in the region. In particular, the role of reptiles in TBP epidemiology remains unknown, despite having been implicated with TBPs of livestock among exported tortoises and lizards. Understanding TBP ecologies, and the potential role of common reptiles, is critical for the development of targeted transmission control strategies for these neglected tropical disease agents. During the wet months (April–May; October–December) of 2012–2013, we surveyed TBP diversity among 4,126 ticks parasitizing livestock and reptiles at homesteads along the shores and islands of Lake Baringo and Lake Victoria in Kenya, regions endemic to diverse neglected tick-borne diseases. After morphological identification of 13 distinct *Rhipicephalus, Amblyomma*, and *Hyalomma* tick species, ticks were pooled (≤8 individuals) by species, host, sampling site, and collection date into 585 tick pools. By supplementing previously established molecular assays for TBP detection with high-resolution melting analysis of PCR products before sequencing, we identified high frequencies of potential disease agents of ehrlichiosis (12.48% *Ehrlichia ruminantium*, 9.06% *Ehrlichia canis*), anaplasmosis (6.32% *Anaplasma ovis*, 14.36% *Anaplasma platys*, and 3.08% *Anaplasma bovis*,), and rickettsiosis (6.15% *Rickettsia africae*, 2.22% *Rickettsia aeschlimannii*, 4.27% *Rickettsia rhipicephali*, and 4.95% *Rickettsia* spp.), as well as *Paracoccus* sp. and apicomplexan hemoparasites (0.51% *Theileria* sp., 2.56% *Hepatozoon fitzsimonsi*, and 1.37% *Babesia caballi*) among tick pools. Notably, we identified *E. ruminantium* in both *Amblyomma* and *Rhipicephalus* pools of ticks sampled from livestock in both study areas as well as in *Amblyomma falsomarmoreum* (66.7%) and *Amblyomma nuttalli* (100%) sampled from tortoises and *Amblyomma sparsum* (63.6%) sampled in both cattle and tortoises at Lake Baringo. Similarly, we identified *E. canis* in rhipicephaline ticks sampled from livestock and dogs in both regions and *Amblyomma latum* (75%) sampled from monitor lizards at Lake Victoria. These novel tick–host–pathogen interactions have implications on the risk of disease transmission to humans and domestic animals and highlight the complexity of TBP ecologies, which may include reptiles as reservoir species, in sub-Saharan Africa.

## Introduction

Tick-borne pathogens (TBPs) are responsible for some of the most serious emerging infectious diseases facing sub-Saharan Africa (SSA) and the rest of the world today (1, 2). In Kenya, TBPs (including viral diseases—arboviruses) like Crimean Congo hemorrhagic fever (CCHF), Dugbe, Kupe, and Hazara, as well as hemoparasites that cause babesiosis, theileriosis, and rickettsiosis, are major impediments to livestock productivity and public health (3–6).

Baringo and Homa Bay counties of Kenya are both characterized by unique land-lake biogeographies with fluctuating fresh water levels of Lakes Baringo and Victoria, respectively. These ecological habitats form shallow lagoons with abundant aquatic and terrestrial biodiversity that include diverse vertebrate host species and disease vectors (7). The interaction of domestic animals (mainly livestock, dogs, chickens, and cats), migratory birds, humans, and wildlife has the potential to facilitate the spread of zoonotic pathogens, including TBPs. Wildlife can act as both sources and maintenance hosts for TBPs (8) that can also be transmitted to livestock and humans (9, 10), causing significant morbidity and mortality (11). For example, East Coast fever (ECF), caused by *Theileria parva*, originates from African buffalo and circulates in cattle, which often succumb to the disease (12, 13).

Recent studies have implicated reptiles as potential reservoirs involved in TBP transmission cycles. *Ehrlichia ruminantium*, considered a pathogen of ruminants responsible for heartwater disease, has been reported among *Amblyomma sparsum* (tortoise tick) sampled from leopard tortoises imported into the United States from Zambia (14). Furthermore, diverse *Borrelia, Rickettsia, Ehrlichia, Anaplasma*, and *Babesia* species have been identified in lizard species in Portugal (15), Australia (16), and the Netherlands (17), as well as in diverse reptiles imported into Japan (18). These studies suggest a possible role of reptiles in the epidemiology of diverse tick-borne diseases, including heartwater, which has not been investigated in African endemic settings.

Increased TBP sharing between wildlife and livestock species may result from human-induced interactions (19, 20). Nomadic and pastoralist lifestyles lead to direct and indirect contact that facilitate exposure and sharing of previously isolated pathogens (21, 22). Wildlife translocations to new habitats have also resulted in outbreaks and mortality among naïve inhabitants, as was the case in cattle when *T. parva*-infected buffalo was translocated to the Highveld of Zimbabwe (23).

In Baringo County, the burden of tick-borne diseases remains largely unknown. The pastoralist communities that inhabit Baringo County plains keep large herds of livestock that are parasitized by an abundant diversity of vector tick species with pathogens that inflict significant economic losses on already drought impoverished populations (24). Interestingly, tick-laden free ranging tortoises that scavenge for food are common in and around homesteads where they interact with humans and livestock. Such domestic–wildlife interactions increase the likelihood of ticks and their pathogens parasitizing different vertebrate taxa, resulting in pathogen spillover.

In Homa Bay County, migratory birds and monitor lizards thrive on popular fishing activities in homesteads along Rusinga Island and may be involved in TBP transmission to humans and livestock. Though information on TBP’s in the region is limited, an over two-decade-old study found *Rhipicephalus appendiculatus* transmitting *T. parva* to be highly prevalent among Zebu cattle grazing along the shores of Lake Victoria in Rusinga Island (25). In neighboring Siaya County, levels of recent human exposure to *Rickettsia felis* infection were found to be exceptionally high (>57%) based on immunoglobulin G (IgG) seropositivity among febrile patients visiting a local health clinic, and *Rickettsia africae* has been isolated in *Amblyomma variegatum* (26, 27). A longitudinal study of cohorts of calves in Busia County found a high prevalence of tick-borne hemoparasites, mainly *Theileria mutans* (71.6%), *Theileria velifera* (62.8%), *Anaplasma* spp. Omatjenne (42.7%), *Anaplasma bovis* (39.9%), *Theileria* sp. (sable) (32.7%), *T. parva* (12.9%), and *Theileria taurotragi* (8.5%) determined by reverse line blot hybridization assay (28). In Uganda, severe anaplasmosis, ECF, and babesiosis were reported a decade ago as causes of livestock morbidity and mortality around the Lake Victoria basin (29). More recently, high prevalences of diverse *Theileria, Anaplasma*, and *Ehrlichia* species have been identified among wildlife hosts in Lake Mburo National Park, Uganda (30) and Laikipia County, Kenya (31), and among ticks sampled in the Shimba Hills National Reserve, Kenya (32).

Intensification of tick and TBP surveillance, disease detection, and control of ticks are critical in informing public health decisions on mitigation, control, and early warning and response strategies in cases of disease outbreaks (33). To gain better insight into the diversity of ticks and TBPs parasitizing livestock, and the potential involvement of cohabitating reptiles in their epidemiology, within the Lake Baringo and Lake Victoria region ecosystems of Kenya, we utilized contemporary molecular biology techniques (32) to screen field-collected ticks sampled along the shores and adjacent islands in these regions. We report the presence and possible circulation of putative tick vectors of TBPs that are etiological agents of ehrlichiosis, anaplasmosis, rickettsiosis, babesiosis, and theileriosis of importance to livestock health and zoonotic diseases in SSA (34, 35).

## Materials and Methods

### Study Locality

A TBP survey was conducted in 2012–2013 along the shores and adjacent islands of Lake Baringo and Lake Victoria in Kenya.

Baringo County is located in the Great Rift Valley, 250 km northwest of Nairobi and covers ~8,655 km^2^ in area. It has an average rainfall of 700 mm and altitude of 700 m above the sea level with average temperatures of 28°C (36). Three indigenous agro-pastoralists communities (Pokot, Tugen, and Njemps) inhabit Baringo County. They rely on livestock (mostly goats as well as cattle, sheep, and donkey) and irrigated crop production along the Perkerra, Molo, and Kerio rivers.

Homa Bay County lies within the Kenyan part of the Lake Victoria basin and covers ~3,155 km^2^ in area. It has an a bimodal rainfall ranging between 250 and 1,650 mm per annum and an altitude of 970 m above the sea level with a mean average temperature range of 17.1 to 34.8°C (37). The “long rains” peak in April and the “short rains” in October. Most inhabitants belong to the Luo and Suba ethnic groups whose main socioeconomic activities are fishing and small-scale mixed farming, which includes keeping of livestock (cattle, sheep, and goats).

### Sampling

Ethical clearance for the study was obtained from the Kenya Medical Research Institute ethics review committee (Approval Ref: non-SSC Protocol #310) and sampling from wildlife was approved by the Kenya Wildlife Service Biodiversity Research and Monitoring committee (Permit Ref: KWS/BRM/5001). Informed oral consent was obtained from village elders on the study activities and from household heads before inclusion of their livestock in the study. Written consent could not be used due to low literacy levels and language barriers that required translation into local languages (Luo and Suba) among most of the community elders and livestock owners, hence oral consent was adopted to all for standardization. The Kenya Wildlife Services, Kenya’s Directorate of Veterinary Services and Ministry of Health, were consulted before the study was initiated, and supervised oral consent and sampling. The oral consent was not documented since tick collection presented minimal risk to the livestock and involved no protocol for which written documentation is normally required. Before sampling, all animals were restrained manually in order to allow for tick collection. To minimize risks to livestock, animal sampling was carried out in a manner that addressed all pertinent animal welfare issues.

Homesteads were surveyed for livestock and reptiles parasitized with ticks in 2012 and 2013 during the wet months of April–May and October–December in both study areas close to human habitation. Those that had recently treated their animals with acaricides were excluded. Ticks were opportunistically sampled and pooled from goats (117), cattle (76), sheep (54), poultry houses (17), and dogs (15) found in selected homesteads, as well as tortoises (*Stigmochelys pardalis*) (18) and monitor lizards (*Varanus niloticus*) (4) that were common to homesteads in Baringo and Homa Bay counties, respectively. Up to 30 ticks were sampled per animal within each homestead and fully engorged ticks were not collected to minimize contamination from vertebrate host nucleic acids during extraction. Animals were manually restrained before plucking of live ticks from their bodies. Crocodiles were excluded because of high risk they pose.

**Figure 1:**
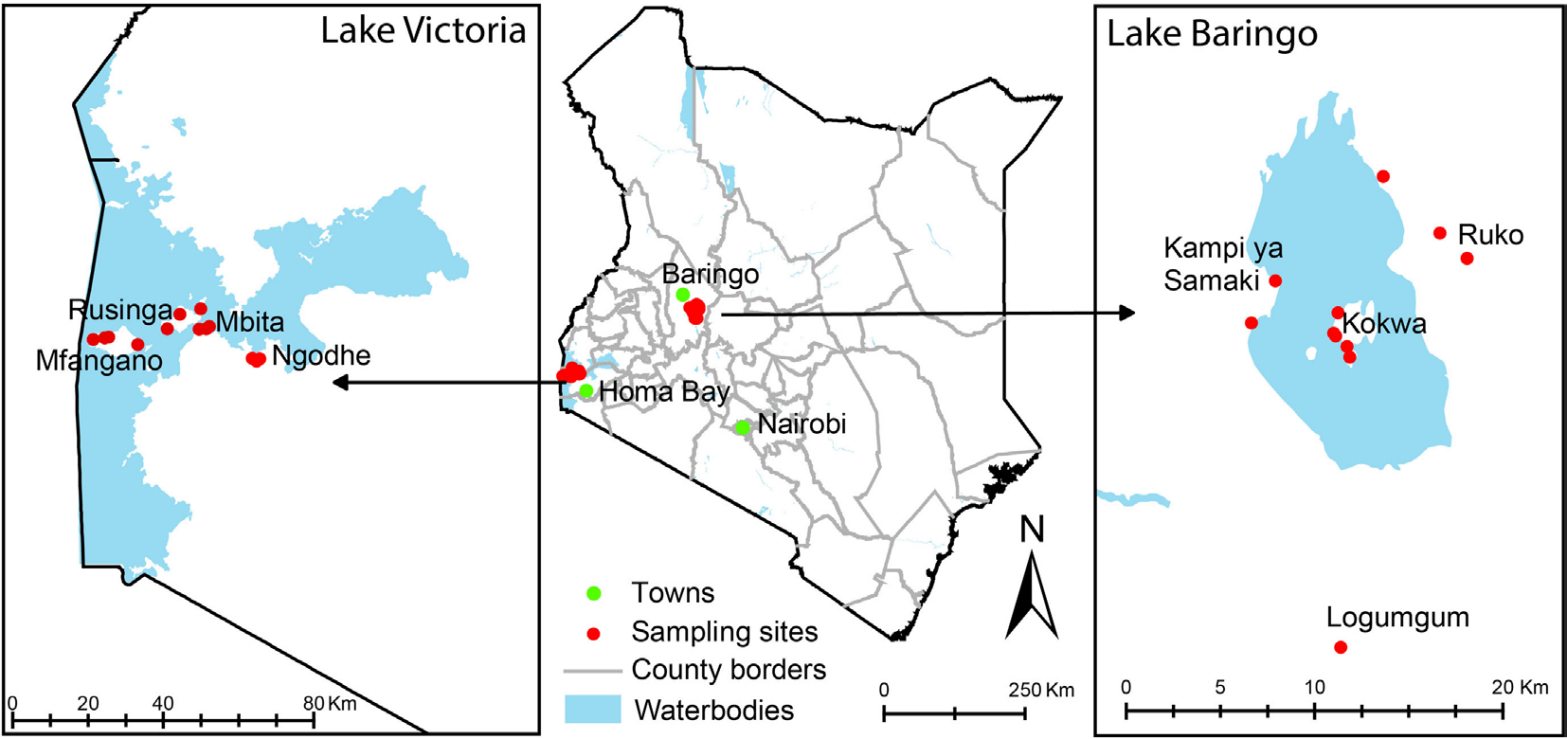
**Map of Kenya showing tick sampling areas in Kenya**. Most of the sampling points were in rural villages where mosquito sampling has previously been described.

Sampling was conducted in Baringo and Homa Bay counties alongside previously described mosquito sampling efforts (38–40) (Figure 1). In Baringo, we sampled in and adjacent to (i) Ruko Wildlife Conservancy, where livestock and humans live in close proximity to wildlife, (ii) Logumgum, a transmission hot spot for the 2006/2007 RVF outbreak (41), (iii) Kokwa, and (iv) Kampi ya Samaki, where locals keep relatively large numbers of livestock (≥20). In Homa Bay County, Rusinga and Mbita areas were chosen due to previous studies in the area that detected the presence of *T. parva* antigens (42) and their association with reduced productivity of Zebu cattle under traditional management (43). Mfangano Island and Ngodhe offer sanctuary to a wide diversity of wild birds that could be important in introductions of ticks to these areas.

After collection, ticks were frozen in liquid nitrogen and transported to the Martin Lüscher Emerging Infectious Diseases Laboratory at the International Centre of Insect Physiology and Ecology in Nairobi, where they were identified morphologically and sorted on a chilled surface (paper towels over −80°C icepacks) under a dissecting microscope (Leica Microsystems, Buffalo Groove, Illinois). Ticks were identified morphologically to species level based on taxonomic keys of the genera *Rhipicephalus* (44), *Amblyomma* (45), *Hyalomma*, and *Argas* (46), developed by Walker and colleagues (47). Tick sorting entailed removal of highly engorged samples to reduce vertebrate host nucleic acids during extraction and pooling into groups of one to eight individuals by species, host, sampling site, and collection date.

### Molecular Identification of TBPs

Tick pools were homogenized for 25 s in 1.5 ml screw-cap tubes filled with zirconia/yttria-stabilized zirconium oxide beads (750 mg of 2.0 mm diameter and 150 mg of 0.1 mm diameter) (Glen Mills, Clifton, NJ, USA) (48), 650 µl of phosphate-buffered saline using a Mini-Beadbeater-16 (BioSpec, Bartlesville, OK, USA). Total nucleic acids were extracted from these homogenates in an automated MagNa Pure 96 extraction system (Roche Diagnostics, Risch-Rotkreuz, Switzerland) using the small volume DNA/viral RNA kits (Roche Diagnostics). TBPs were detected and characterized by PCR followed by high-resolution melting (HRM) analyses. All gene fragments were amplified in an HRM capable RotorGene Q thermo cycler (QIAGEN, Hannover, Germany) to a final volume of 10 µl using HOT FIREPol EvaGreen HRM Mix (Solis BioDyne, Tartu, Estonia) and primers listed in Table 1. For identification of *Ehrlichia* and *Anaplasma*, we utilized previously described primers (49) of a short 16S rRNA amplicon (< 200 bp) and unresolved samples were subsequently analyzed by amplification and sequencing of longer (> 300bp) 16S rRNA fragments using the newly designed primer pairs, “*Anaplasma* long 16S rRNA Fwd/Rev” and “*Ehrlichia* long 16S rRNA Fwd/Rev” (Table 1). For *Rickettsia*, we utilized the *rpm*E-tRNA^fMet^ intergenic spacer typing (50). The apicomplexan hemoparasites, *Theileria, Babesia* and *Hepatozoon*, were amplified and resolved using previously described primers specific to the 18S ribosomal gene (Table 1) (51).

**Table 1:** **Primers used for detection of TBPs**.

Pathogen/gene target	Primer pair	Amplicons size (bp)	Reference sequence	Primer coordinates	Citation
*Anaplasma* short 16S rRNA	Fwd: GGGCATGTAGGCGGTTCGGT	~200	KJ410254	491–510	Tokarz et al. (49)
	Rev: TCAGCGTCAGTACCGGACCA			675–656	
*Anaplasma* long 16S rRNA	Fwd: CGGTGGAGCATGTGGTTTAATTC	~330		853–875	Mwamuye et al. (32)
	Rev: CGRCGTTGCAACCTATTGTAGTC			1,183–1,161	
*Ehrlichia* short 16S rRNA	Fwd: CGTAAAGGGCACGTAGGTGGACTA	~200	NR_074155	507–530	Tokarz et al. (49)
	Rev: CACCTCAGTGTCAGTATCGAACCA			701–678	
*Ehrlichia* long 16S rRNA	Fwd: GCAACCCTCATCCTTAGTTACCA	~400		1,045–1,067	Mwamuye et al. (32)
	Rev: TGTTACGACTTCACCCTAGTCAC			1,439–1,417	
*Rickettsia*/*ompB*	For: GTAAAATTACCGGTAAGGGTTATAGC	~200	CP001612	1,020,788–1,020,813	Tokarz et al. (49)
	Rev: ATACAAAGTGCTAATGCAACTGGG			1,020,984–1,020,961	
*Rickettsia rpmE*/tRNA^fMet^	For: TCAGGTTATGAGCCTGACGA	175–402		130,181–130,205	Zhu et al. (50)
	Rev: TTCCGGAAATGTAGTAAATCAATC			130,522–130,503	
*Theileria/Babesia* 18S rRNA	Fwd: GAGGTAGTGACAAGAAATAACAATA	~500	HQ684067	330–354	Gubbels et al. (51)
	Rev: TCTTCGATCCCCTAACTTTC			832–813	

The thermal cycling conditions used for amplification were as follows: initial denaturation at 95°C for 15 min, followed by 10 cycles of 94°C for 20 s, step-down annealing from 63.5°C decreasing by 1°C per cycle for 25 s, and primer extension at 72°C for 30 s; then 25 cycles of denaturation at 94°C for 25 s, annealing at 50.5 for 20 s, and extension at 72°C for 30 s, followed by a final extension at 72°C for 7 min. Following PCR, HRM profiles of amplicons were obtained through gradual increase in temperature from 75 to 90°C at 0.1°C/2 s increments. Changes in fluorescence with time (dF/dT) were plotted against changes in temperature (°C). Positive samples/amplicons were detected by observation of HRM curves and peaks. To identify the specific pathogen sequences associated with each unique HRM profiles, representative samples with single peaks for each of the profiles were purified with ExoSAP-IT PCR Product Cleanup kit (Affymetrix, Santa Clara, CA, USA) to remove unincorporated dNTPs and PCR primers before sequencing, which was outsourced from Macrogen (South Korea).

### Phylogenetic Analysis

The returned sequences were edited and aligned, using the MAFFT (52) plugin in Geneious software version 8.1.4 (created by Biomatters) (53), with closely related sequences revealed by querying the GenBank nr database using the Basic Local Alignment Search Tool (54). Study sequences that were >200 bp were submitted to GenBank. From multiple alignments, some pathogens could not be fully characterized to species and were classified based on their genus. After visualizing neighbor-joining phylogenetic trees (55) of the alignments, constructed within Geneious software, we constructed comparable maximum likelihood phylogenetic trees of the alignments, using PHYML v. 3.0 (56). The phylogenies employed the Akaike information criterion for automatic model selection and tree topologies were estimated using nearest neighbor interchange improvements over 1,000 bootstrap replicates. Phylogenetic trees were depicted using FIGTREE (57). *Bacillus subtilis* rpmE/tRNA^fMet^ (GenBank accession CP010434) and *Hemolivia stellata* 18S rRNA (GenBank accession KP881349) sequences were used as outgroups for the *Rickettsia* and apicomplexan hemoparasite phylogenies, respectively. The *Ehrlichia*/*Anaplasma* 16S rRNA phylogeny was midpoint rooted with the *Paracoccus* 16S rRNA sequences as no suitable outgroup sequences were available in public databases. Midpoint rooting is appropriate for this phylogeny since the *Paracoccus* clade is distant to the *Ehrlichia/Anaplasma* clade, ensuring consistency with outgroup rooting procedures (58).

## Results

### Tick Species Sampled

A total of 585 tick pools comprised of 4,126 ticks of 14 species were collected and analyzed from both study areas. We sampled more ticks (80.47%) in Baringo County (3,320 ticks in 456 tick pools) (Table 2), which had higher numbers of livestock per household (>20), than in Homa Bay County (806 ticks in 129 tick pools) (Table 3), which had fewer (<5) livestock per household, from diverse vertebrate hosts. In both study areas, most ticks were from goats, which were more heavily parasitized than the other animals sampled (Tables 2 and 3).

**Table 2:** **Ticks sampled from different host species in four study areas of Baringo County**.

Study area	Tick species	Pools	N (%)	Cattle (%)	Goats (%)	Sheep (%)	Dogs (%)	Tortoises (%)	Poultry (%)
Kampi Ya Samaki	*Rh. pravus*	51	401 (12.07)	82 (2.47)	186 (5.6)	95 (2.86)	38 (1.14)		
	*Ar. persicus*	34	234 (7.04)						234 (7.04)
	*Rh. pulchellus*	31	204 (6.14)	46 (1.38)	98 (2.95)	58 (1.74)	2 (0.06)		
	*Rh. evertsi evertsi*	17	133 (4)	24 (0.72)	66 (1.98)	38 (1.14)	5 (0.15)		
	*Am. variegatum*	5	33 (0.99)	19 (0.57)	5 (0.15)	9 (0.27)			
	*Am. gemma*	4	17 (0.51)	8 (0.24)	6 (0.18)	3 (0.09)			
	*Am. sparsum*	2	13 (0.39)	13 (0.39)					
	*Am. nuttalli*	1	6 (0.18)					6 (0.18)	
Ruko Conservancy	*Rh. pravus*	31	235 (7.07)	52 (1.56)	128 (2.85)	55 (1.65)			
	*Rh. evertsi evertsi*	28	219 (6.59)	59 (1.77)	46 (1.38)	68 (2.04)	46 (1.38)		
	*Rh. pulchellus*	27	209 (6.29)	114 (3.43)	46 (1.38)	30 (0.9)	19 (0.57)		
	*Hy. truncatum*	13	101 (3.04)	52 (1.56)	14 (0.42)	35 (1.05)			
	*Am. variegatum*	8	52 (1.56)	41 (1.23)	6 (0.18)	5 (0.15)			
	*Hy. rufipes*	7	47 (1.41)	28 (0.84)	7 (0.21)	6 (0.18)			6 (0.18)
	*Am. gemma*	5	36 (1.08)	18 (0.54)	11 (0.33)	7 (0.21)			
	*Am. sparsum*	4	32 (0.96)					32 (0.96)	
	*Am. nuttalli*	2	16 (0.48)					16 (0.48)	
	*Rh. praetextatus*	8	15 (0.45)	10 (0.3)	5 (0.15)				
Logumgum	*Rh. pravus*	97	731 (22.01)	161 (4.85)	304(9.15)	202 (6.08)	64 (1.92)		
	*Am. variegatum*	26	187 (5.83)	121 (3.64)	23 (0.69)	43(1.29)			
	*Rh. evertsi evertsi*	14	110 (3.31)	25 (0.75)	24 (0.72)	15 (0.45)	46 (1.38)		
	*Am. gemma*	6	47 (1.41)	19(0.57)	5(0.15)	23 (0.69)		41 (1.23)	
	*Am. falsomarmoreum*	6	41 (1.23)					30 (0.9)	
	*Am. sparsum*	5	30 (0.9)						
Kokwa	*Rh. pravus*	16	123 (3.7)	1 (0.03)	84 (2.53)	38 (1.14)			
	*Rh. evertsi evertsi*	4	26 (0.78)	4 (0.12)	8 (0.24)	9 (0.27)	5 (0.15)		
	*Am. gemma*	4	22 (0.66)	2 (0.06)	12 (0.36)	8 (0.24)			
	Total	456	3,320	899 (27.07)	1,084 (32.56)	747 (22.5)	225 (6.77)	125 (3.76)	240 (7.22)

*Percentages are out of the total number of ticks sampled. N, number of ticks sampled*.

**Table 3:** **Ticks sampled from different host species in four study areas of Homa Bay County**.

Study Area	Tick species	Pools	N (%)	Cattle (%)	Goats (%)	Sheep (%)	Dogs (%)	Monitor lizards (%)
Ngothe	*Rh. pulchellus*	9	61 (7.56)	24 (2.97)	23 (2.85)	14(1.73)		
	*Rh. evertsi evertsi*	8	45 (5.58)	3 (0.37)	30 (2.72)	4 (0.49)	8 (2.23)	
	*Am. variegatum*	4	18 (2.23)	9(1.11)	4 (0.49)	5 (0.62)		
	*Hy. truncatum*	3	12 (1.48)	9(1.11)		3 (0.37)		
Mbita	*Rh. pravus*	13	83 (10.29)	13(1.61)	38(4.71)	28 (2.47)	4 (0.49)	
	*Am. gemma*	9	67 (8.31)	51 (6.32)	12(1.48)	4 (0.49)		
	*Rh. evertsi evertsi*	7	40 (4.96)	11 (1.36)	18(2.23)	6 (0.74)	5 (0.62)	
	*Am. Variegatum*	5	32 (3.97)	16 (1.98)	6 (0.74)	10(1.24)		
	*Rh. pulchellus*	1	6 (0.74)	6 (0.74)				
Mfangano	*Rh. pravus*	14	94 (11.66)	18 (2.23)	37 (4.59)	27 (2.35)	12(1.48)	
	*Am. variegatum*	6	45 (5.58)	25 (3.1)	14(1.73)	6 (0.74)		
	*Rh. pulchellus*	8	41 (5.08)	9 (1.11)	19(2.35)	13(1.61)		
	*Rh. evertsi evertsi*	5	26 (3.22)	5 (0.62)	14(1.73)	4 (0.49)	3 (0.37)	
Rusinga	*Rh. pravus*	14	101 (12.53)	15 (1.86)	57 (7.07)	15(1.86)	14(1.73)	
	*Rh. pulchellus*	7	50 (6.2)	12 (1.48)	26 (3.22)	11 (1.36)	1 (0.12)	
	*Rh. appendiculatus*	5	29 (3.59)	25 (3.1)	4 (0.49)			
	*Am. latum*	4	27 (3.34)					27 (3.34)
	*Rh. evertsi evertsi*	6	23 (2.85)	4 (0.49)	11 (1.36)	2 (0.24)	6 (0.74)	
	*Hy. truncatum*	1	6 (0.74)	6 (0.74)				
	Total	129	806	261 (32.38)	307 (38.08)	152(18.85)	53 (6.57)	27 (3.34)

*Percentages are out of the total number of ticks sampled. N, Number of ticks sampled*.

In Baringo County, 12 species were sampled from goats, sheep, cattle, poultry houses, dogs, and free ranging tortoises (Table 2), 11 of which were hard ticks (Family; *Ixodidae*) and one of which was a soft tick species (*Argas persicus*, 7.04%) sampled from poultry houses in Kampi ya Samaki. *Rhipicephalus pravus* (44.88%) sampled from domestic ruminants and dogs was the most frequent tick species identified, while *Amblyomma falsomarmoreum* (1.23%) and *Amblyomma nuttalli* (0.66%) sampled from free ranging tortoises at Kampi ya Samaki were the least frequent tick species identified.

In Homa Bay County, eight hard tick species were sampled from goats, sheep, cattle, dogs, and monitor lizards (Table 3). *Rhipicephalus pravus* (34.49%), sampled from livestock and dogs, was the most frequent species identified, while *Hyalomma truncatum* (2.23%) sampled from livestock was least frequent. *Rhipicephalus appendiculatus* (3.59%), a known vector for ECF, and *Amblyomma* (*Aponomma*) *latum* (3.34%), sampled from monitor lizards, were common only on Rusinga Island.

### TBPs Identified

**Figure 2:**
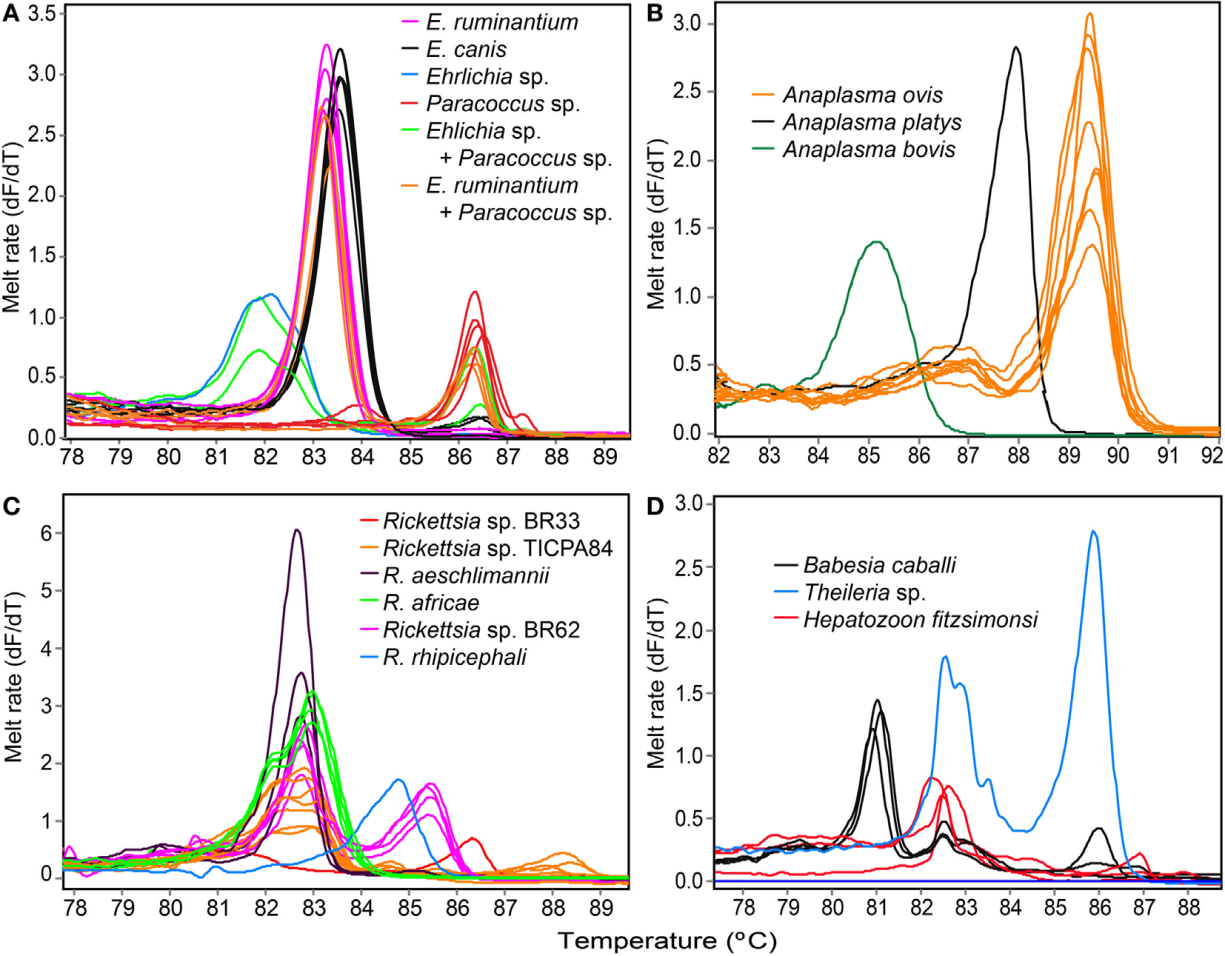
**Melting rate profiles of tick-borne pathogens in field collected tick samples**. PCR amplicon melt rates are represented as change in fluorescence with increasing temperature (dF/dT) of **(A)** *Ehrlichia* spp. and *Paracoccus* sp. 16S rRNA, **(B)** *Anaplasma* 16S rRNA, **(C)** *Rickettsia rpmE*/tRNA^fMet^, and **(D)** apicomplexan hemoparasite 18S rRNA gene segments.

**Figure 3:**
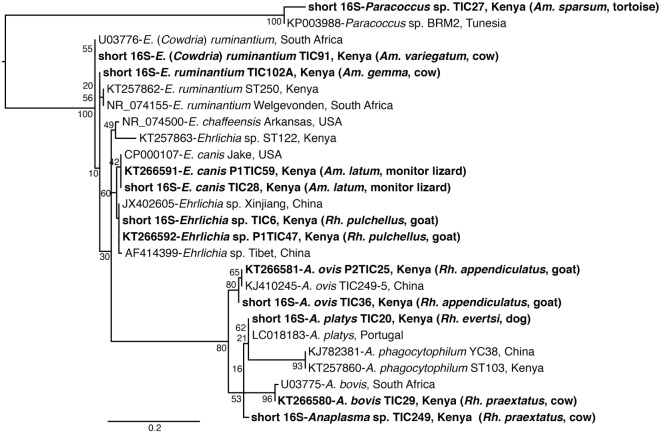
**Maximum likelihood phylogenetic analysis of 16S rRNA gene fragments of *Anaplasma, Ehrlichia*, and *Paracoccus* sequences identified with related sequences**. GenBank accession numbers, species identifications, isolates, and country of origin are indicated for each 16S rRNA gene sequence. Sequences from this study are in bold with tick and vertebrate host species associated with the study isolates indicated in brackets. Bootstrap values at the major nodes are of percentage agreement among 1,000 replicates. The branch length scale represents substitutions per site.

**Figure 4:**
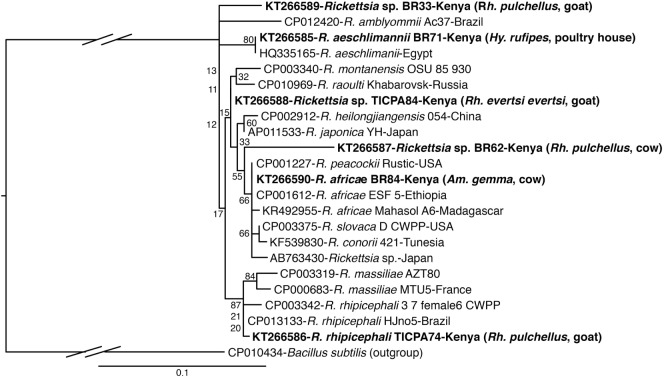
**Maximum likelihood phylogenetic analysis of rpmE/tRNA^fMet^ gene fragments of *Rickettsia* sequences identified with related sequences**. GenBank accession numbers, species identifications, isolates, and country of origin are indicated for each 16S rRNA gene sequence. Sequences from this study are in bold with tick and vertebrate host species associated with the study isolates indicated in brackets. Bootstrap values at the major nodes are of percentage agreement among 1,000 replicates. The branch length scale represents substitutions per site. The gaps indicated in the branches to the *Bacillus subtilis* outgroup represent 1.2 substitutions per site.

**Figure 5:**
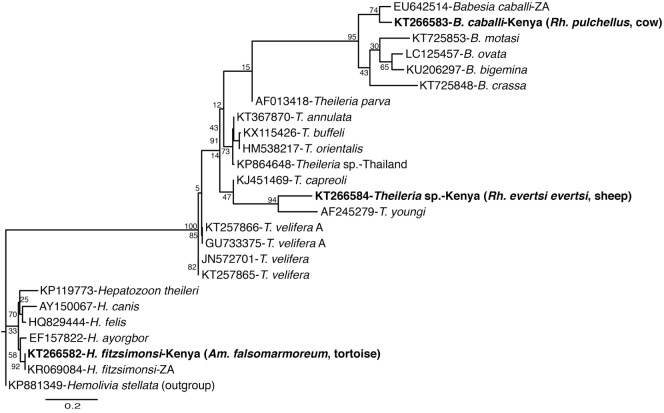
**Maximum likelihood phylogenetic analysis of 18S rRNA gene fragments of Apicomplexa (*Hepatozoon, Babesia*, and *Theileria*) sequences identified with related sequences**. GenBank accession numbers, species identifications, isolates, and country of origin are indicated for each 16S rRNA gene sequence. Sequences from this study are in bold with tick and vertebrate host species associated with the study isolates indicated in brackets. Bootstrap values at the major nodes are of percentage agreement among 1,000 replicates. The branch length scale represents substitutions per site.

Tick-borne pathogen gene fragments with distinct HRM profiles (Figure 2) and representative sequences sharing ≥96% identity with a recognized TBP species, or ≥90% with a TBP genus on GenBank (Table 4), were detected in tick pools sampled in Baringo and Homa Bay counties (Table 5) from diverse individual vertebrate hosts (Table 6). In Baringo County, we detected sequences of agents of livestock or canine ehrlichiosis (*E. ruminantium, Ehrlichia canis*, and *Ehrlichia* sp.) (Figure 2A), anaplasmosis (*Anaplasma ovis, Anaplasma platys*, and *A. bovis*) (Figure 2B), rickettsiosis (*Rickettsia aeschlimannii, Rickettsia rhipicephali, Rickettsia africae*, and *Rickettsia* spp.) (Figure 2C), as well as *Babesia caballi, Hepatozoon fitzsimonsi, Theileria* sp. (Figure 2D), and *Paracoccus* sp. (Figure 2A; Table 5). However, in Homa Bay County, we only detected *E. ruminantium, E. canis, A. ovis*, and *A. platys* (5). The maximum likelihood phylogenies of all sequences obtained among previously characterized, closely related TBPs are represented in Figures 3–5.

**Table 4:** **Nucleic acid sequence identities of tick-borne pathogens detected in Baringo and Homa Bay counties**.

Pathogen detected	Tick species	Study areas	Reference GenBank Accessions	Percentage identity (17 November 2016 *e*-value)	Locus	Sequence length, bp (GenBank Accession)
*Anaplasma bovis*	*Am. gemma, Am. variegatum, Rh. evertsi evertsi*, and *Hy. truncatum*	Logumgum and Ruko	U03775	99 (1e−135)	Short 16S	185
	*Hy. rufipes, Rh. praetextat\us*			99 (1e−163)	Long 16S	320 (KT266580)
*A. ovis*	*Am. variegatum, Rh. pulchellus, Am. gemma*, and *Rh. appendiculatus*	Logumgum, Kampi Ya Samaki, and Rusinga	KJ410245	99 (1e−135)	Short 16S	185
				100 (8e−170)	Long 16S	328 (KT266581)
*A. platys*	*Rh. evertsi, Rh. pulchellus*, and *Rh. pravus*	Ngothe, Mfangano, Kampi ya Samaki, and Kokwa	LC018183	100 (5e−162)	Short 16S	185
*Ehrlichia ruminantium*	*Am. gemma, Am. variegatum, Am. sparsum*, and *Am. evertsi evertsi*	Ruko and Logumgum	NR_074155	99 (1e−94)	Short 16S	194
*E*. (*Cowdria*) *ruminantium*	*Am. gemma, Am. variegatum*, and *Rh. evertsi evertsi*	Ngothe, Mfangano, and Mbita	U03776	99 (6e−93)	Short 16S	194
*E. canis*	*Rh. pravus, Rh. evertsi evertsi, Rh. pulchellus*, and *Am. latum*	Logumgum, Rusinga, and Mbita	CP000107	100 (3e−96) 100 (0.0)	Short 16S	194
					Long 16S	555 (KT266591)
*Ehrlichia* sp. (Tibet/Xinjiang)	*Rh. pulchellus, Am. gemma*, and *Am. variegatum*	Ruko	AF414399, JX402605	98 (3e−96)	Short 16S	194
	*Rh. evertsi evertsi, Rh. pulchellus*, and *Rh. praetextatus*	Ruko and Kokwa Island		100 (0.0)	Long 16S	384 (KT266592)
*Paracoccus* sp.	*Am. variegatum, Am. gemma, Am. sparsum*, and *Am. falsomarmoreum*	Logumgum	KP003988	97 (6e−88)	Short 16S	151
*Rickettsia africae*	*Am. gemma, Am. variegatum, Am. sparsum*, and *Rh. evertsi evertsi*	Kampi Ya Samaki, Ruko, and Logumgum	CP001612	100 (3e−143)	tRNA	280 (KT266590)
*R. rhipicephali*	*Rh. evertsi evertsi* and *Rh. pulchellus*	Kampi ya Samaki, Ruko	CP013133	99 (2e−176)	tRNA	343 (KT266586)
*R. aeschlimannii*	*Hy. truncatum, Hy. rufipes*, and *Rh. pulchellus*	Kokwa, Logumgum, and Kampi ya Samaki	HQ335165	100 (1e−178)	tRNA	344 (KT266585)
*Rickettsia* sp. BR62	*Rh. pulchellus* and *Rh. evertsi evertsi*	Ruko	AP011532	94 (7e−115)	tRNA	281 (KT266587)
*Rickettsia* sp. TICPA84	*Am. gemma* and *Rh. evertsi evertsi*	Logumgum and Kokwa	CP013133	99 (2e−171)	tRNA	343 (KT266588)
*Rickettsia* sp. BR33	*Rh. pulchellus*	Ruko	KR492955	97 (1e−52)	tRNA	354 (KT266589)
*Babesia caballi*	*Rh. pulchellus*	Kampi ya Samaki	EU642514	98 (1e−122)	18S	255 bp (KT266583)
*Theileria* sp.	*Rh. evertsi evertsi*	Logumgum	AF245279	90 (3e−140)	18S	402 (KT266584)
*Hepatozoon fitzsimonsi*	*Am. falsomarmoreum* and *Am. sparsum*	Logumgum and Ruko	KR069084	100 (0.0)	18S	438 (KT266582)

**Table 5:** **Tick-borne pathogens (TBPs) isolated from tick pools collected in Baringo and Homa Bay counties**.

TBP	*Rh. pravus*	*Rh. evertsi evertsi*	*Rh. pulchellus*	*Am. variegatum*	*Am. gemma*	*Rh. appendiculatus*	*Hy. truncatum*	*Am. sparsum*	*Rh. praetextatus*	*Hy. rufipes*	*Am. falsomarmoreum*	*Am. nuttalli*	*Am. latum*	Total
**Baringo pools (N)**	195	63	58	39	19		13	11	8	7	6	3		
*A. bovis*		6 (9.52%)	3 (5.17%)	4 (10.25%)	1 (5.26%)		1 (7.69%)		1 (25%)	1 (14.28%)				18 (3.95%)
*A. ovis*			5 (8.62%)	22 (56.51%)	7 (36.84%)									34 (7.45%)
*A. platys*	**45 (23.07%)**	16 (23.81%)	4 (6.89%)											64 (14.03%)
*E. ruminantium*		**6 (9.52%)**		23 (58.97%)	13 (68.42%)			7 (63.6%)			**4 (66.7%)**	**3 (100%)**		56 (12.28%)
*E. canis*	**13 (6.67%)**	20 (31.74%)	11 (18.96%)											44 (9.64%)
*Ehrlichia sp*.		13 (20.63%)	9 (15.51%)	3 (7.69%)	7 (36.84%)				1 (12.5%)					33 (7.23%)
*Paracoccus sp*.				**1 (2.56%)**	**8 (42.1%)**			**9 (81.81%)**			**2 (33.3%)**			20 (4.38%)
*R. africae*		3 (4.76%)	**3 (5.17%)**	16 (41.02%)	14 (73.68%)									36 (7.89%)
*R. rhipicephalii*		4 (6.34%)	21 (36.2%)											25 (5.48%)
*R. aeschlimannii*			2 (3.44%)				6 (46.15%)			5 (71.41%)				13 (2.85%)
*Rickettsia spp*.		**11 (17.46%)**	**12 (20.68%)**		**6 (31.57%)**									29 (6.35%)
*Babesia caballi*			8 (13.79%)											8 (1.75%)
*Theileria* sp.		3 (4.76%)												3 (0.66%)
*H. fitzsimonsi*								9 (81.81%)			5 (83.3%)			15 (3.28%)
**Homa Bay pools (N)**	41	26	25	15	9	5							4	
*A. ovis*						3 (60%)								3 (2.32%)
*A. platys*	**7 (17.07%)**	7 (26.92%)	6 (24%)											20 (15.5%)
*E. ruminantium*		**5 (19.23%)**		6 (35.29%)	6 (66.67%)									17 (17.52%)
*E. canis*			6 (24%)										**3 (75%)**	9 (6.97%)

*Likely novel tick-TBP associations are highlighted in bold. N, number of tick pools. Percentages are out of the number of specific tick species sampled*.

**Table 6:** **Vertebrate hosts from which ticks with tick-borne pathogens (TBPs) were isolated in the two study regions**.

TBP	Cattle (%)	Goat (%)	Sheep (%)	Dog (%)	Tortoise	Monitor lizard (%)
**Baringo County**						
N (vertebrate hosts)	45	87	33	9	18	0
*Anaplasma bovis*	8 (17.8)	6 (6.8)	3 (9.1)			
*A. ovis*	7 (15.6)	5 (5.7)	10 (30.3)			
*A. platys*		17 (19.5)		9 (100)		
*Ehrlichia ruminantium*	17 (37.8)	14 (16.1)	3 (9.1)		12 (66.7)	
*E. canis*		15 (17.2)		9 (100)		
*Ehrlichia* sp.	11 (24.4)	6 (6.9)	6 (18.2)	2 (13.3)		
*Paracoccus* sp.	**5 (11.1)**	**8 (9.2)**	**3 (9.1)**		**3 (16.7)**	
*Rickettsia africae*	17 (37.8)	6 (6.8)	11 (33.3)			
*R. rhipicephali*	8 (17.7)	4 (3.4)	5 (15.2)	**2 (22.2)**		
*R. aeschlimannii*	5 (11.1)	2 (2.3)	6 (18.2)			
*Rickettsia* sp. BR62		**5 (5.7)**				
*Rickettsia* sp. TICPA84	**3 (6.7)**	**2 (2.3)**	**5 (15.2)**	**2 (22.2)**		
*Rickettsia* sp. BR33		**2 (2.3)**				
*Babesia caballi*	8 (17.7)					
*Theileria* sp.	3 (6.7)					
*Hepatozoon fitzsimonsi*					14 (77.8)	
**Homa Bay County**						
N (vertebrate hosts)	31	30	21	7	0	4
*A. ovis*	1 (3.2)	1 (3.3)	1 (4.8)			
*A. platys*	4(12.9)	2 (6.6)	3 (14.3)	4 (57.1)		
*E*. (*Cowdria*) *ruminantium*	9 (29.0)	4 (13.3)				
*E. canis*	1 (3.2)			4 (57.1)		**4 (100)**

*Likely novel vertebrate host-TBP associations are highlighted in bold. N, number of vertebrate hosts*.

*Percentages are out of the number of specific vertebrate host species sampled*.

Among *Ehrlichia* sequences identified, *E. ruminantium* sequences from Baringo shared 99% nucleotide sequence identity with GenBank accession NR_074155 (59), whereas those from Homa Bay County were more similar (99% identity) to an *E*. (*Cowdria*) *ruminantium* sequence (GenBank accession U03776) (60) (Table 4). In both sampling areas, these were detected in *Am. variegatum, Amblyomma gemma*, and *Rhipicephalus evertsi evertsi* tick pools (Table 5) from livestock and dogs (Tables 2, 3 and 6). Surprisingly, *E. ruminantium* was also detected in *Am. falsomarmoreum* (four pools) and *Am. nuttalli* (three pools) ticks (Table 5) sampled from tortoises (Tables 2 and 6) and, more importantly, *Am. sparsum* ticks (Table 5) sampled from both tortoises (five pools) and cattle (two pools) in Logumgum and Kampi ya Samaki, respectively, in Baringo County (Tables 2 and 6). *Ehrlichia canis* sequences (submitted GenBank accession KT266591) (Table 4) were detected in *Rh. pravus, Rh. evertsi evertsi*, and *Rhipicephalus pulchellus* tick pools (Table 5) sampled from livestock and domestic dogs of Logumgum in Baringo County (Tables 2 and 6), *Rh. pulchellus* ticks (Table 5) sampled from cattle in Mbita (Tables 3 and 6), and notably, *Am*. (*aponomma*) *latum* ticks (Table 5) parasitizing monitor lizards in Rusinga Island (Tables 3 and 6) of Homa Bay County. *Ehrlichia* sp. sequences (submitted GenBank accession KT266592) sharing 98–100% identity with *Ehrlichia* spp. isolates from Tibet and Xinjiang, China (GenBank accessions AF414399, JX402605) (61) (Table 4) were detected in *Am. gemma* (7 pools), *Am. variegatum* (3 pools), *Rh. evertsi evertsi* (13 pools), *Rh. pulchellus* (9 pools), and *Rh. praetextatus* (1 pool) ticks (Table 5) sampled from livestock in Ruko, Kokwa Island, and Logumgum areas of Baringo County (Tables 2 and 6).

We also amplified 195 bp *Paracoccus* sp. sequences using the “*Ehrlichia* short 16S rRNA” primers (Table 4) in *Am. variegatum* (one pool), *Am. sparsum* (nine pools), *Am. falsomarmoreum* (two pools), and *Am. gemma* (eight pools) (Table 5) ticks sampled from livestock and tortoises (Tables 2 and 6). There were instances in which we detected both *Paracoccus* sp. and *E. ruminantium* within single tick pools as illustrated by double HRM peaks (Figure 2A). Six pools of *Am. sparsum* and four of *Am. variegatum* had both *E. ruminantium* and *Paracoccus* sp., while three pools of *Am. variegatum* had a mixed infection of *Ehrlichia* sp. and *Paracoccus* sp. (Figure 2A).

The *A. ovis* sequences (submitted GenBank accession KT266581) (Table 4) were detected in *Am. variegatum* (22 pools), *Am. gemma* (7 pools), and *Rh. pulchellus* (5 pools) ticks (Table 5) parasitizing livestock in Logumgum and Kampi ya Samaki of Baringo County (Tables 2 and 6) and in *Rh. appendiculatus* (3 pools) (Table 5) parasitizing goats and cattle in Rusinga Island of Homa Bay County (Tables 3 and 6). *Anaplasma platys* sequences (Table 4) were detected from *Rhipicephalus* tick pools, including *Rh. evertsi evertsi* (15 pools), *Rh. pravus* (45 pools), and *Rh. pulchellus* (4 pools) (Tables 5 and 6) sampled from domesticated dogs in Ruko Conservancy, Kampi ya Samaki, Ngodhe, Mbita, Mfangano, and Rusinga Island study areas (Tables 2, 3 and 6). *Anaplasma bovis* sequences (submitted GenBank accession KT266580) (Table 4) were detected in 18 pools of *Amblyomma* ticks*. Hyalomma* and *Rhipicephalus* ticks (Table 5) sampled from livestock of Logumgum and Ruko study areas (Tables 2 and 6).

The *R. aeschlimannii* pathogen sequences (submitted GenBank accession KT266585) (Table 4) were detected in *Hy. truncatum* (6 pools), *Hyalomma rufipes* (5 pools), and *Rh. pulchellus* (2 pools) ticks (Table 5) sampled from livestock in Logumgum, Kampi ya Samaki, and Kokwa Island (Tables 2 and 6)*. Rickettsia rhipicephali* sequences (submitted GenBank accession KT266586) (Table 4) were amplified in *Rh. evertsi evertsi* (4 pools) and *Rh. pulchellus* (21 pools) ticks (Table 5) sampled from goats, sheep, cattle, and dogs at Kampi ya Samaki, Logumgum, and Ruko Conservancy of Baringo County (Tables 2 and 6). *Rickettsia africae* sequences (submitted GenBank accession KT266590) (Table 4) were detected in *Am. gemma* (14 pools), *Am. variegatum* (16 pools), *Am. sparsum* (3 pools), and *Rh. evertsi evertsi* (3 pools) (Table 5) parasitizing livestock in Kampi ya Samaki, Logumgum, and Ruko Conservancy of Baringo County (Tables 2 and 6). We also obtained three novel *Rickettsia* spp. sequences from Baringo County tick pools. The *Rickettsia* sp. BR62 (submitted GenBank accession KT266587) and BR33 (submitted GenBank accession KT266589) sequences (Table 4) were detected in *Rh. pulchellus* (12 pools) and *Rh. evertsi evertsi* (9 pools) ticks (Table 5) sampled from goats in Ruko Conservancy (Tables 2 and 6). *Rickettsia* sp. TICPA84 sequences (submitted GenBank accession KT266588) were detected in *Am. gemma* (six pools) from Logumgum and *Rh. evertsi evertsi* (three pools) from livestock and dogs (Tables 5 and 6) *in* Kokwa Island (Table 2).

Among apicomplexan hemoparasites, we detected *B. caballi* sequences (submitted GenBank accession KT266583) (Table 4) in *Rh. pulchellus* (eight pools) ticks (Table 5) parasitizing cattle in Kampi ya Samaki, Baringo County (Tables 2 and 6). *Hepatozoon fitzsimonsi* sequences (submitted GenBank accession KT266582) (Table 4) were amplified in *Am. sparsum* (nine pools) and *Am. falsomarmoreum* (five pools) ticks (Table 5) sampled from free ranging tortoises in Baringo County (Tables 2 and 6). *Theileria* sp. sequences (submitted GenBank accession KT266584) (Table 4) were also amplified in *Rh. evertsi evertsi* (three pools) ticks (Table 5) parasitizing cattle at Logumgum in Baringo County (Tables 2 and 6).

## Discussion

We sampled 13 tick species that are potential vectors of ehrlichiosis, anaplasmosis, rickettsiosis, theileriosis, and babesiosis from livestock, poultry houses, and reptiles in domestic surroundings of Baringo and Homa Bay Counties. Most tick species were taken from multiple host taxa, except for *Hy. truncatum* that was restricted to cattle, *Ar. persicus* that was restricted to poultry, *Am. latum* that was restricted to monitor lizards, and *Am. nuttalli* and *Am. falsomarmoreum* that were both restricted to tortoises. Overall, we identified 14 TBPs, most from multiple tick species and predominantly from Baringo County samples. These included novel *Ehrlichia* sp., *Rickettsia* sp., *Paracoccus* sp., and *Theileria* sp. sequences that warrant further investigations into their potential pathogenicity. Most interestingly, *Am. sparsum* ticks infected with *E. ruminantium*, the causative agent of heartwater ehrlichiosis, were sampled from both tortoises and cattle. The complex pathogen–tick–host relationships presented here are important to public health in mitigating TBP transmission and possible associated disease outbreaks in these foci, as well as other areas of Kenya, with wider geographical implications.

Heartwater ehrlichiosis is an important rickettsial disease of wildlife and livestock ruminants in SSA, with considerable economic impact (62). Unlike past studies, which found *E. ruminantium* to be specific to *Amblyomma* tick species (63, 64), we also isolated the pathogen from *Rh. evertsi evertsi* sampled from livestock in both study locations, though we cannot rule out that this may have come from livestock blood meals. *Amblyomma variegatum* Fabricius 1794 is the most common and widely distributed *Amblyomma* tick species of livestock in SSA and is a very important vector of the *E. ruminantium* in cattle (65). *Amblyomma gemma* Dönitz 1909 sampled from livestock in both study areas was once thought to be less important as a disease vector, but has since been linked with the transmission of a number of pathogens (65, 66). Laboratory studies have also shown that *Am. gemma* can transmit *E. ruminantium* from infected African buffalo to sheep (67). In this study, we detected *E. ruminantium* in 58.97 and 35.29% of *Am. variegatum* tick pools and in 68.42 and 66.67% of *Am. gemma* tick pools sampled in Baringo and Homa Bay Counties, respectively, suggesting very high infection rates. However, due to the pooled approach used to identify TBP’s, these infection percentages do not provide a true estimate of infection rates.

Most importantly, *E. ruminantium* was also detected in *Am. sparsum* samples from both cattle and tortoises in Baringo as well as in *Am. falsomarmoreum and Am. nuttalli* found exclusively on tortoises. The occurrence of *E. ruminantium* has previously been reported among *Am. sparsum* that were sampled from tortoises imported into the United States from Zambia (14). *Amblyomma nuttalli* and *Am. falsomarmoreum* have both been associated with tortoises before (68, 69), but no report that we are aware of has implicated them with harboring or transmitting of agents of heartwater ehrlichiosis. Our findings suggest that *E. ruminantium* may be potentially transmitted between cattle and tortoises by *Am. sparsum* ticks and within tortoise populations by *Am. nuttalli* and *Am. falsomarmoreum* ticks. Similarly, we found *E. canis*, the causative agent of canine ehrlichiosis, in *Am. latum* ticks taken from monitor lizards in Homa Bay County. These pathogen–tick–host associations suggest complex transmission dynamics in the epidemiology of heartwater in Baringo County and canine ehrlichiosis in Homa Bay County that potentially involve reptilian reservoir hosts that are rarely considered in epidemiological studies of these pathogens.

From domestic ruminants and dogs, *Rh. pravus* Dönitz 1910 was the most frequent tick parasite sampled in Baringo and Homa Bay counties, followed by *Rh. evertsi evertsi* and *Rh. pulchellus*. Each of these three tick species were found parasitizing dogs, with pools infected with canine anaplasmosis (*A. platys*) and ehrlichiosis (*E. canis*), the latter only in Baringo. *Rhipicephalus pravus* is considered the most common tick species among domestic and wildlife animals in Kenya (70–72), but has rarely been implicated in pathogen transmission. These findings potentially incriminate *Rh. pravus* in the transmission of both *A. platys* and *E. canis*. Further studies to determine its vector competence will be critical to understanding its role in TBP transmission.

We detected *A. bovis* in multiple species of each of the tick genera (*Rhipicephalus, Amblyomma*, and *Hyalomma*) parasitizing livestock in Baringo County, but none in Homa Bay. The highest rates of *A. bovis* detection occurred in *Rh. praetextatus* Gerstäcker 1873, a three-host tick species that was sampled from cattle and goats in Baringo County. *Rhipicephalus praetextatus* from Ngorongoro crater of Tanzania has been implicated in the transmission of *A. marginale*, the principal agent of bovine anaplasmosis (73). Similarly, we found *A. ovis* in both *Rhipicephalus* and *Amblyomma* ticks in Baringo, but only in *Rh. appendiculatus* sampled from livestock in Homa Bay County. In contrast, in Baringo County, where no *Rh. appendiculatus* were sampled, *A. ovis* occurred most frequently in *Am. variegatum*. These *Anaplasma* infections are rarely associated with clinical symptoms in livestock, but may affect livestock health synergistically during coinfections with other livestock disease agents (74, 75) in Baringo County.

Sequences of spotted fever group (SFG) Rickettsiae (*R. africae, R. aeschlimannii*, and *R. rhipicephalii*) and three novel *Rickettsia* spp. were identified in tick (*Rh. evertsi evertsi* and *Rh. pulchellus*) samples from livestock and dogs only in Baringo County; no *Rickettsia* were identified in Homa Bay County, despite the fact that in neighboring Siaya County, a high prevalence of *R. africae*, the etiological agent of African tick bite fever, was previously identified among *Am. variegatum* parasitizing domestic ruminants (27). In this study, *Rickettsia africae* was particularly prevalent in *Am. gemma* (73.68%) and *Am. variegatum* (41.02%) tick pools, but was also found in *Rh. evertsi evertsi* (4.67%) and *Rh. pulchellus* (5.17%) tick pools sampled from livestock. We detected *R. aeschlimannii* predominantly in *Hyalomma* tick pools (*Hy. truncatum* and *Hy. marginatum rufipes*) (55.0%) and incidentally in *Rh. pulchellus* tick pools (3.44%). In *Hy. truncatum* tick pools that were sampled from cattle, sheep, and goats, we detected both *A. bovis* and *R. aeschlimannii*. Separate pools of *Hy. marginatum rufipes* parasitizing livestock were also positive for *A. bovis* and *R. aeschlimannii*. *Rickettsia aeschlimannii* has previously been isolated from *Hy. truncatum* parasitizing camels in the Kano area of Nigeria (76). Though SFG rickettsiosis caused by this TBP has been rarely reported in SSA, it has been reported in humans in Algeria (77) and has also been found in *Am. variegatum* ticks in western Kenya (27). The likely role of *Hyalomma* ticks as reservoirs for *R. aeschlimannii* (78) makes these ticks of particular importance in the epidemiology of SFG rickettsiosis in East Africa and warrants further attention. *Rickettsia rhipicephalii*, as well two of the novel *Rickettsia* spp. (BR62, BR33), were confined to rhipicephaline (*Rh. pulchellus* and *Rh. eversti evertsi*) ticks. A third *Rickettsia* sp. (TICPA84) was found in *Am. gemma* (31.57%) and *Rh. evertsi evertsi* (4.76%) tick pools from livestock and dogs. While these findings confirm higher prevalences of *Rickettsia* spp. in specific *Amblyomma* and *Hyalomma* species, their occurrence in *Rhipicephalus* ticks, perhaps opportunistic, should be considered in the transmission ecology of these TBPs.

From livestock parasitizing ticks in Baringo, we only detected one novel *Theileria* sp. sequence in *Rh. evertsi evertsi* ticks and *B. caballi* in *Rh. pulchellus* ticks from cattle, despite the fact that diverse *Theileria* (79) and *Babesia* (6) species antigens have been found in livestock surveillance studies in different regions in Kenya. Nonetheless, 29 (0.7% of sampled ticks) *Rh. appendiculatus* Neumann 1901, a vector of *T. parva* (80), were sampled from livestock of Homa Bay County, but not in Baringo. This was contrary to a previous study done over two decades previously, which found *Rh. appendiculatus* to be highly prevalent among Zebu cattle grazing along the shores of Lake Victoria in Rusinga Island (25). This change may be an indication of tick control efforts, most likely with acaricides, or difference in sampling period. Although we detected *A. ovis* in three *Rh. appendiculatus* tick pools, we did not find *T. parva*, indicating a possible absence of *T. parva* or extremely low prevalence within our study sites.

Additionally, we found both *H. fitzsimonsi* and *Paracoccus* sp. bacteria in *Am. falsomarmoreum* and *Am. sparsum* ticks from tortoises in Baringo. In South Africa, tortoises have recently been documented to harbor concurrent parasitic infection with *H. fitzsimonsi* (81). *Paracoccus* sp. bacteria, also identified in pools of *Am. variegatum* and *Am. gemma* ticks sampled from livestock in Baringo County, was first reported in a population of *Am. cajennense* from South America in 2012, but it is still unknown if *Paracoccus* infection in ticks is a group of pathogenic rhodobacteraceae or simply plays a role in tick physiology (82). Nonetheless, our findings demonstrate that primers targeting specific *Ehrlichia* 16S rRNA gene fragment could also be used for detection of *Paracoccus* species.

While only one to three TBPs were identified in most tick species, none were identified in *Ar. persicus* sampled from poultry houses, and more than five TBPs were identified in *Rh. evertsi evertsi, Rh. Pulchellus*, and *Am. gemma* tick pools, all of which have been widely implicated in TBP transmission (65, 66, 83–85). Although all 28 pools of *Ar. persicus* were negative for TBPs, its large population in poultry houses and frequent blood feeding behavior has been linked with nuisance, severe anemia, paralysis, and toxicosis in poultry (86). Furthermore, more TBPs were isolated from Baringo County ticks, suggesting more complex and higher transmission rates in the region, where higher numbers of livestock per household (>20) are kept, compared to the fewer (<5) livestock that are kept by Homa Bay County households.

This study reports the presence, possible circulation, and putative transmission sources of TBPs that are etiological agents of ehrlichiosis, anaplasmosis and rickettsiosis, and hemoparasites of importance to livestock health in the study areas. Among these, we found surprisingly high infection rates of *E. ruminantium* and *E. canis*, agents of livestock and canine ehrlichiosis, respectively, in ticks sampled from reptilian hosts, suggesting their likely role as reservoir species in the epidemiology of these TBPs. Proper and rapid diagnoses that include analyses of these TBPs in livestock and their proximal wildlife species are important in mitigating disease burden and possible outbreaks in these areas and the rest of SSA.

## Ethics Statement

This study was carried out in accordance with the recommendations of the Kenya Medical Research Institute (KEMRI), Wildlife Service (KWS), Directorate of Veterinary Services and Ministry of Health. The protocol was approved by the KEMRI ethics review committee (Approval Ref: non-SSC Protocol #310) and the KWS Biodiversity Research and Monitoring committee (Permit Ref: KWS/BRM/5001).

## Author Contributions

DO, DM, and JV designed the study. DO, EK, and YA conducted the fieldwork. DO, MM, and DOO conducted the laboratory work. DO and JV conducted the analysis and drafted the manuscript; BM, MM, and DM contributed to the interpretation of the data. All the authors contributed to the manuscript editing and approved the final manuscript.

## Conflict of Interest Statement

The authors declare that the research was conducted in the absence of any commercial or financial relationships that could be construed as a potential conflict of interest.

## References

[1] Dunster L Dunster M Ofula V Beti D Kazooba-Voskamp F Burt F First documentation of human Crimean-Congo hemorrhagic fever, Kenya. Emerg Infect Dis (2002) 8:1005–6. 10.3201/eid0809.010510 12194785 PMC2732535

[2] Parola P Paddock CD Raoult D. Tick-borne rickettsioses around the world: emerging diseases challenging old concepts. Clin Microbiol Rev (2005) 18:719–56. 10.1128/CMR.18.4.719-756.2005 16223955 PMC1265907

[3] Parola P. Rickettsioses in sub-Saharan Africa. Ann N Y Acad Sci (2006) 1078:42–7. 10.1196/annals.1374.005 17114679

[4] Sang R Lutomiah J Koka H Makio A Chepkorir E Ochieng C Crimean-Congo hemorrhagic fever virus in Hyalommid ticks, northeastern Kenya. Emerg Infect Dis (2011) 17:1502–5. 10.3201/eid1708.102064 21801635 PMC3381575

[5] Lwande OW Venter M Lutomiah J Michuki G Rumberia C Gakuya F Whole genome phylogenetic investigation of a West Nile virus strain isolated from a tick sampled from livestock in north eastern Kenya. Parasit Vectors (2014) 7:542. 10.1186/s13071-014-0542-2 25430727 PMC4255437

[6] Kiara H Jennings A Bronsvoort B Handel IG Mwangi ST Mbole-Kariuki M A longitudinal assessment of the serological response to *Theileria parva* and other tick-borne parasites from birth to one year in a cohort of indigenous calves in Western Kenya. Parasitology (2014) 141:1289–98. 10.1017/S003118201400050X 24838078 PMC4113304

[7] Minakawa N Sonye G Dida GO Futami K Kaneko S. Recent reduction in the water level of Lake Victoria has created more habitats for *Anopheles funestus*. Malar J (2008) 7:119. 10.1186/1475-2875-7-119 18598355 PMC2490699

[8] Kock RA What is this infamous “wildlife/livestock disease interface?” A review of current knowledge for the African continent. In: Osofsky SA, editor. Conservation and Development Interventions at the Wildlife/Livestock Interface – Implications for Wildlife, Livestock and Human Health. Switzerland: IUCN (2005). p. 1–13.

[9] Dantas-Torres F Chomel BB Otranto D. Ticks and tick-borne diseases: a one health perspective. Trends Parasitol (2012) 28:437–46. 10.1016/j.pt.2012.07.003 22902521

[10] Gortazar C Ferroglio E Hofle U Frolich K Vicente J Diseases shared between wildlife and livestock: a European perspective. Eur J Wildl Res (2007) 53:241–56. 10.1007/s10344-007-0098-y

[11] Uilenberg G. International collaborative research: significance of tick-borne hemoparasitic diseases to world animal health. Vet Parasitol (1995) 57:19–41. 10.1016/0304-4017(94)03107-8 7597784

[12] Bengis R Kock R Fischer J. Infectious animal diseases: the wildlife/livestock interface. Rev Sci Tech (2002) 21:53–66. 10.20506/rst.21.1.1322 11974630

[13] Olwoch JM Reyers B Engelbrecht FA Erasmus BFN Climate change and the tick-borne disease, Theileriosis (East Coast fever) in sub-Saharan Africa. J Arid Environ (2008) 72:108–20. 10.1016/j.jaridenv.2007.04.003

[14] Burridge MJ Simmons LA Simbi BH Peter TF Mahan SM. Evidence of *Cowdria ruminantium* infection (heartwater) in *Amblyomma sparsum* ticks found on tortoises imported into Florida. J Parasitol (2000) 86:1135–6. 10.2307/3284836 11128494

[15] De Sousa R Lopes de Carvalho I Santos AS Bernardes C Milhano N Jesus J Role of the lizard *Teira dugesii* as a potential host for *Ixodes ricinus* tick-borne pathogens. Appl Environ Microbiol (2012) 78(10):3767–9. 10.1128/AEM.07945-11 22407681 PMC3346372

[16] Whiley H Custance G Graves S Stenos J Taylor M Ross K *Rickettsia* detected in the reptile tick *Bothriocroton hydrosauri* from the lizard *Tiliqua rugosa* in South Australia. Pathogens (2016) 5(2):E41. 10.3390/pathogens5020041 27338482 PMC4931392

[17] Tijsse-Klassen E Fonville M Reimerink JH Spitzen-van der Sluijs A Sprong H. Role of sand lizards in the ecology of Lyme and other tick-borne diseases in the Netherlands. Parasit Vectors (2010) 3:42. 10.1186/1756-3305-3-42 20470386 PMC2890652

[18] Andoh M Sakata A Takano A Kawabata H Fujita H Une Y Detection of *Rickettsia* and *Ehrlichia* spp. in ticks associated with exotic reptiles and amphibians imported into Japan. PLoS One (2015) 10(7):e0133700. 10.1371/journal.pone.0133700 26207382 PMC4514593

[19] Miller RS Farnsworth ML Malmberg JL Diseases at the livestock–wildlife interface: status, challenges, and opportunities in the United States. Prev Vet Med (2013) 110:119–32. 10.1016/j.prevetmed.2012.11.021 23254245 PMC7127607

[20] Wiethoelter AK Beltrán-Alcrudo D Kock R Mor SM Global trends in infectious diseases at the wildlife–livestock interface. Proc Natl Acad Sci U S A (2015) 112:9662–7. 10.1073/pnas.1422741112 26195733 PMC4534210

[21] Daszak P Cunningham AA Hyatt AD. Anthropogenic environmental change and the emergence of infectious diseases in wildlife. Acta Trop (2001) 78:103–16. 10.1016/S0001-706X(00)00179-0 11230820

[22] Maxwell JF Lea B-F Davies TJ The study of parasite sharing for surveillance of zoonotic diseases. Environ Res Lett (2013) 8:015036 10.1088/1748-9326/8/1/015036 PMC7106949 32288780

[23] Latif AA Hove T Kanhai GK Masaka S. Buffalo-associated *Theileria parva*: the risk to cattle of buffalo translocation into the Highveld of Zimbabwe. Ann N Y Acad Sci (2002) 969:275–9. 10.1111/j.1749-6632.2002.tb04392.x 12381605

[24] Homewood K Lewis J Impact of drought on pastoral livestock in Baringo, Kenya 1983–85. J Appl Ecol (1987) 24:615–31. 10.2307/2403897

[25] Punyua DK Latif AA Nokoe S Capstick PB. Tick (Acari: Ixodidae) infestations on Zebu cattle in western Kenya: seasonal dynamics of four species of ticks on traditionally managed cattle. J Med Entomol (1991) 28:630–6. 10.1093/jmedent/28.5.630 1941930

[26] Maina AN Knobel DL Jiang J Halliday J Feikin DR Cleaveland S *Rickettsia felis* infection in febrile patients, Western Kenya, 2007-2010. Emerg Infect Dis (2012) 18:328–31. 10.3201/eid1802.111372 22304807 PMC3310467

[27] Maina AN Jiang J Omulo SA Cutler SJ Ade F Ogola E High prevalence of *Rickettsia africae* variants in *Amblyomma variegatum* ticks from domestic mammals in rural Western Kenya: implications for human health. Vector Borne Zoonotic Dis (2014) 14:693–702. 10.1089/vbz.2014.1578 25325312 PMC4208559

[28] Njiiri NE Bronsvoort BMC Collins NE Steyn HC Troskie M Vorster I The epidemiology of tick-borne haemoparasites as determined by the reverse line blot hybridization assay in an intensively studied cohort of calves in western Kenya. Vet Parasitol (2015) 210:69–76. 10.1016/j.vetpar.2015.02.020 25858115 PMC4427107

[29] Rubaire-Akiiki C Okello-Onen J Nasinyama G Vaarst M Kabagambe EK Mwayi W The prevalence of serum antibodies to tick-borne infections in Mbale District, Uganda: the effect of agro-ecological zone, grazing management and age of cattle. J Insect Sci (2004) 4:8. 10.1673/031.004.0801 15861224 PMC528868

[30] Oura CA Tait A Asiimwe B Lubega GW Weir W. *Theileria parva* genetic diversity and haemoparasite prevalence in cattle and wildlife in and around Lake Mburo National Park in Uganda. Parasitol Res (2011) 108(6):1365–74. 10.1007/s00436-010-2030-8 20827491

[31] Ghai RR Mutinda M Ezenwa VO. Limited sharing of tick-borne hemoparasites between sympatric wild and domestic ungulates. Vet Parasitol (2016) 226:167–73. 10.1016/j.vetpar.2016.07.005 27514903

[32] Mwamuye MM Kariuki E Omondi D Kabii J Odongo D Masiga D Novel *Rickettsia* and emergent tick-borne pathogens: a molecular survey of ticks and tick-borne pathogens in Shimba Hills National Reserve, Kenya. Ticks Tick Borne Dis (2017) 8:208–18. 10.1016/j.ttbdis.2016.09.002 28011185

[33] de la Fuente J Kocan KM Almazan C Blouin EF. Targeting the tick-pathogen interface for novel control strategies. Front Biosci (2008) 13:6947–56. 10.2741/3201 18508707

[34] Crump JA Morrissey AB Nicholson WL Massung RF Stoddard RA Galloway RL Etiology of severe non-malaria febrile illness in Northern Tanzania: a prospective cohort study. PLoS Negl Trop Dis (2013) 7:e2324. 10.1371/journal.pntd.0002324 23875053 PMC3715424

[35] Tigoi C Lwande O Orindi B Irura Z Ongus J Sang R. Seroepidemiology of selected arboviruses in febrile patients visiting selected health facilities in the lake/river basin areas of Lake Baringo, Lake Naivasha, and Tana River, Kenya. Vector Borne Zoonotic Dis (2015) 15:124–32. 10.1089/vbz.2014.1686 25700043 PMC4340645

[36] Kipkorir EC Analysis of rainfall climate on the Njemps Flats, Baringo District, Kenya. J Arid Environ (2002) 50:445–58. 10.1006/jare.2001.0917

[37] Mati BM The influence of climate change on maize production in the semi-humid–semi-arid areas of Kenya. J Arid Environ (2000) 46:333–44. 10.1006/jare.2000.0699

[38] Omondi D Masiga DK Ajamma YU Fielding BC Njoroge L Villinger J. Unraveling host-vector-arbovirus interactions by two-gene high resolution melting mosquito bloodmeal analysis in a Kenyan wildlife-livestock interface. PLoS One (2015) 10(7):e0134375. 10.1371/journal.pone.0134375 26230507 PMC4521840

[39] Onchuru TO Ajamma YU Burugu M Kaltenpoth M Masiga D Villinger J Chemical parameters and bacterial communities associated with larval habitats of *Anopheles, Culex* and *Aedes* mosquitoes (Diptera: Culicidae) in Western Kenya. Int J Trop Insect Sci (2016) 36(3):146–60. 10.1017/S1742758416000096

[40] Ajamma YU Villinger J Omondi D Salifu D Onchuru TO Njoroge L Composition and genetic diversity of mosquitoes (Diptera: Culicidae) on islands and mainland shores of Kenya’s Lakes Victoria and Baringo. J Med Entomol (2016) 53(6):1348–63. 10.1093/jme/tjw102 27402888 PMC5106823

[41] Sang R Kioko E Lutomiah J Warigia M Ochieng C O’Guinn M Rift Valley fever virus epidemic in Kenya, 2006/2007: the entomologic investigations. Am J Trop Med Hyg (2010) 83:28–37. 10.4269/ajtmh.2010.09-0319 20682903 PMC2913497

[42] Morzaria SP Musoke AJ Latif AA Recognition of *Theileria parva* antigens by field sera from Rusinga Island, Kenya. Kenya Vet (1988) 12:8.

[43] Latif AA Rowlands GJ Punyua DK Hassan SM Capstick PB An epidemiological study of tick-borne diseases and their effects on productivity of zebu cattle under traditional management on Rusinga Island, Western Kenya. Prev Vet Med (1995) 22:169–81. 10.1016/0167-5877(94)00408-B

[44] Walker JB Keirans JE Horak IG The Genus Rhipicephalus (Acari, Ixodidae): A Guide to the Brown Ticks of the World. Cambridge: Cambridge University Press (2000).

[45] Walker JB Olwage A The tick vectors of *Cowdria ruminatum* (Ixodoidea: Ixodidae, genus *Amblyomma*) and their distribution. Onderstepoort J Vet Res (1987) 54(3):353–79. 3329325

[46] Walker JB The Ixodid Ticks of Kenya: A Review of Present Knowledge of Their Hosts and Distribution. London: Commonwealth Institute of Entomology (1974).

[47] Walker AR Bouattour A Camicas J-L Estrada-Peña A Horak IG Latif AA Ticks of Domestic Animals in Africa: A Guide to Identification of Species. Edinburgh, Scotland: Bioscience Reports (2003).

[48] Crowder CD Rounds MA Phillipson CA Picuri JM Matthews HE Halverson J Extraction of total nucleic acids from ticks for the detection of bacterial and viral pathogens. J Med Entomol (2010) 47:89–94. 10.1093/jmedent/47.1.89 20180313 PMC2837073

[49] Tokarz R Kapoor V Samuel JE Bouyer DH Briese T Lipkin WI. Detection of tick-borne pathogens by MassTag polymerase chain reaction. Vector Borne Zoonotic Dis (2009) 9:147–52. 10.1089/vbz.2008.0088 18800864 PMC2976645

[50] Zhu Y Fournier PE Ogata H Raoult D. Multispacer typing of *Rickettsia prowazekii* enabling epidemiological studies of epidemic typhus. J Clin Microbiol (2005) 43:4708–12. 10.1128/JCM.43.9.4708-4712.2005 16145131 PMC1234059

[51] Gubbels JM de Vos AP van der Weide M Viseras J Schouls LM de Vries E Simultaneous detection of Bovine *Theileria* and *Babesia* species by reverse line blot hybridization. J Clin Microbiol (1999) 37:1782–9. 10325324 10.1128/jcm.37.6.1782-1789.1999 PMC84950

[52] Katoh K Standley DM. MAFFT multiple sequence alignment software version 7: improvements in performance and usability. Mol Biol Evol (2013) 30:772–80. 10.1093/molbev/mst010 23329690 PMC3603318

[53] Kearse M Moir R Wilson A Stones-Havas S Cheung M Sturrock S Geneious basic: an integrated and extendable desktop software platform for the organization and analysis of sequence data. Bioinformatics (2012) 28(12):1647–9. 10.1093/bioinformatics/bts199 22543367 PMC3371832

[54] Altschul SF Gish W Miller W Myers EW Lipman DJ Basic local alignment search tool. J Mol Biol (1990) 15(3):403–10. 10.1016/S0022-2836(05)80360-2 2231712

[55] Saitou N Nei M. The neighbor-joining method: a new method for reconstructing phylogenetic trees. Mol Biol Evol (1987) 4(4):406–25. 3447015 10.1093/oxfordjournals.molbev.a040454

[56] Guindon S Dufayard JF Lefort V Anisimova M Hordijk W Gascuel O. New algorithms and methods to estimate maximum-likelihood phylogenies: assessing the performance of PHYML 3.0. Syst Biol (2010) 59:307–21. 10.1093/sysbio/syq010 20525638

[57] Drummond AJ Rambaut A. BEAST: Bayesian evolutionary analysis by sampling trees. BMC Evol Biol (2007) 7:214. 10.1186/1471-2148-7-214 17996036 PMC2247476

[58] Hess PN De Moraes Russo CA An empirical test of the midpoint rooting method. Biol J Linn Soc (2007) 92:669–74. 10.1111/j.1095-8312.2007.00864.x PMC7110036 32287391

[59] Collins NE Liebenberg J de Villiers EP Brayton KA Louw E Pretorius A The genome of the heartwater agent *Ehrlichia ruminantium* contains multiple tandem repeats of actively variable copy number. Proc Natl Acad Sci U S A (2005) 102(3):838–43. 10.1073/pnas.0406633102 15637156 PMC545511

[60] Allsopp M Visser ES du Plessis JL Vogel SW Allsopp BA. Different organisms associated with heartwater as shown by analysis of 16S ribosomal RNA gene sequences. Vet Parasitol (1997) 71(4):283–300. 10.1016/S0304-4017(97)00012-5 9299697

[61] Wen B Jian R Zhang Y Chen R. Simultaneous detection of *Anaplasma marginale* and a new *Ehrlichia* species closely related to *Ehrlichia chaffeensis* by sequence analyses of 16S ribosomal DNA in *Boophilus microplus* ticks from Tibet. J Clin Microbiol (2002) 40(9):3286–90. 10.1128/JCM.40.9.3286-3290.2002 12202567 PMC130830

[62] Mukhebi AWT Chamboko CJ O’Callaghan TF Peter RL Kruska GF Medley SM An assessment of the economic impact of heartwater (*Cowdria ruminantium* infection) and its control in Zimbabwe. Prev Vet Med (1999) 39:173–89. 10.1016/S0167-5877(98)00143-3 10327437

[63] Kelly PJ Lucas H Yowell C Beati L Dame J Urdaz-Rodriguez J *Ehrlichia ruminantium* in *Amblyomma variegatum* and domestic ruminants in the Caribbean. J Med Entomol (2011) 48:485–8. 10.1603/ME10172 21485394

[64] Trout Fryxell RT DeBruyn JM. Correction: the microbiome of *Ehrlichia*-infected and uninfected lone star ticks (*Amblyomma americanum*). PLoS One (2016) 11:e0155559. 10.1371/journal.pone.0155559 26751816 PMC4709196

[65] Ngumi PN Rumberia RM Williamson SM Sumption KJ Lesan AC Kariuki DP. Isolation of the causative agent of heartwater (*Cowdria ruminantium*) from three *Amblyomma* species in eight districts of Kenya. Vet Rec (1997) 140:13–6. 10.1136/vr.140.1.13 9004475

[66] Wesonga FD Mukolwe SW Rurangirwa F. *Cowdria ruminantium* identified in *Amblyomma gemma* using a DNA probe pCS20. Rev Elev Med Vet Pays Trop (1993) 46:179–81. 8134629

[67] Wesonga FD Mukolwe SW Grootenhuis J. Transmission of *Cowdria ruminantium* by *Amblyomma gemma* from infected African buffalo (*Syncerus caffer*) and eland (*Taurotragus oryx*) to sheep. Trop Anim Health Prod (2001) 33:379–90. 10.1023/A:1010539705913 11556617

[68] Horak IG McKay IJ Henen BT Heyne H Hofmeyr MD De Villiers AL. Parasites of domestic and wild animals in South Africa. XLVII. Ticks of tortoises and other reptiles. Onderstepoort J Vet Res (2006) 73:215–27. 10.4102/ojvr.v73i3.148 17058444

[69] Nowak M The international trade in reptiles (Reptilia) – the cause of the transfer of exotic ticks (Acari: Ixodida) to Poland. Vet Parasitol (2010) 169:373–81. 10.1016/j.vetpar.2010.01.006 20153933

[70] Burgdorfer W Ormsbee RA Schmidt ML Hoogstraal H. A search for the epidemic typhus agent in Ethiopian ticks. Bull World Health Organ (1973) 48:563–9. 4204491 PMC2482933

[71] Wanzala W Okanga S. Ticks (Acari: Ixodidae) associated with wildlife and vegetation of Haller park along the Kenyan coastline. J Med Entomol (2006) 43:789–94. 10.1093/jmedent/43.5.789 17017210

[72] Kariuki EK Penzhorn BL Horak IG. Ticks (Acari: Ixodidae) infesting cattle and African buffaloes in the Tsavo conservation area, Kenya. Onderstepoort J Vet Res (2012) 79:1–4. 10.4102/ojvr.v79i1.437 23327329

[73] Fyumagwa RD Simmler P Meli ML Hoare R Hofmann-Lehmann R Lutz H. Prevalence of *Anaplasma marginale* in different tick species from Ngorongoro Crater, Tanzania. Vet Parasitol (2009) 161:154–7. 10.1016/j.vetpar.2008.12.018 19201099

[74] Hornok S Edelhofer R Foldvari G Joachim A Farkas R. Serological evidence for *Babesia canis* infection of horses and an endemic focus of *B. caballi* in Hungary. Acta Vet Hung (2007) 55:491–500. 10.1556/AVet.55.2007.4.8 18277708

[75] Thumbi SM de C Bronsvoort BM Poole EJ Kiara H Toye P Parasite co-infections show synergistic and antagonistic interactions on growth performance of East African zebu cattle under one year. Parasitology (2013) 140:1789–98. 10.1017/S0031182013001261 24001119 PMC3829697

[76] Kamani J Baneth G Apanaskevich DA Mumcuoglu KY Harrus S Molecular detection of *Rickettsia aeschlimannii* in *Hyalomma* spp. ticks from camels (*Camelus dromedarius*) in Nigeria, West Africa. Med Vet Entomol (2015) 29:205–9. 10.1111/mve.12094 25565180

[77] Mokrani N Parola P Tebbal S Dalichaouche M Aouati A Raoult D *Rickettsia aeschlimannii* infection, Algeria. Emerg Infect Dis (2008) 14:1814–5. 10.3201/eid1411.071221 18976583 PMC2630723

[78] Matsumoto K Parola P Brouqui P Raoult D *Rickettsia aeschlimannii* in *Hyalomma* ticks from Corsica. Eur J Clin Microbiol Infect Dis (2004) 23:732–4. 10.1007/s10096-004-1190-9 15309667

[79] Moumouni PF Aboge GO Terkawi MA Masatani T Cao S Kamyingkird K Molecular detection and characterization of *Babesia bovis, Babesia bigemina, Theileria* species and *Anaplasma marginale* isolated from cattle in Kenya. Parasit Vectors (2015) 8:496. 10.1186/s13071-015-1106-9 26420543 PMC4589125

[80] Norval RAI Perry BD Young A The Epidemiology of Theileriosis in Africa. London, UK: Academic Press (1992).

[81] Cook CA Smit NJ Davies AJ. Hemoproteids (Apicomplexa: Haemoproteidae) from South African tortoises (Cryptodira: Testudinidae). J Parasitol (2010) 96:1168–72. 10.1645/GE-2527.1 21158631

[82] Machado-Ferreira E Piesman J Zeidner NS Soares CA. A prevalent alpha-proteobacterium *Paracoccus* sp. in a population of the cayenne ticks (*Amblyomma cajennense*) from Rio de Janeiro, Brazil. Genet Mol Biol (2012) 35:862–7. 10.1590/S1415-47572012005000067 23271948 PMC3526095

[83] Mediannikov O Diatta G Fenollar F Sokhna C Trape JF Raoult D. Tick-borne rickettsioses, neglected emerging diseases in rural Senegal. PLoS Negl Trop Dis (2010) 4(9):e821. 10.1371/journal.pntd.0000821 20856858 PMC2939048

[84] Wanzala W Ondiaka SN. Tick-borne lymphadenopathy-like condition in an African woman in Kenya. J Res Med Sci (2013) 18:918–21. 24497868 PMC3897081

[85] Kumsa B Socolovschi C Almeras L Raoult D Parola P. Occurrence and genotyping of *Coxiella burnetii* in ixodid ticks in Oromia, Ethiopia. Am J Trop Med Hyg (2015) 93:1074–81. 10.4269/ajtmh.14-0758 26392155 PMC4703278

[86] Rosenstein M Paralysis in chickens caused by larvae of the poultry tick, *Argas persicus*. Avian Dis (1976) 20:407–9. 10.2307/1589281 938388

